# Isolated Polycystic Liver Disease: An Unusual Cause of Recurrent Variceal Bleed

**DOI:** 10.1155/2018/2902709

**Published:** 2018-06-04

**Authors:** Mohammad Saud Khan, Zubair Khan, Toseef Javaid, Jamal Akhtar, Abdelmoniem Moustafa, Amos Lal, Abhinav Tiwari, Mohammad Taleb

**Affiliations:** ^1^Department of Internal Medicine, University of Toledo Medical Center, Toledo, OH, USA; ^2^Department of Internal Medicine, Saint Vincent Hospital, Worcester, MA, USA; ^3^Division of Pulmonary, Critical Care and Sleep Medicine, University of Toledo Medical Center, Toledo, OH, USA

## Abstract

Isolated polycystic liver disease is a rare disorder. Majority of the patients with isolated polycystic liver disease are asymptomatic with incidental detection of liver cysts on imaging studies done for other purposes. Minority of patients develop symptoms which are mostly secondary to enlarging cysts size and hepatomegaly. Rarely, these patients develop portal hypertension and can present with its clinical manifestations and consequences in the form acute variceal bleeding or recurrent ascites. We present a rare case of 67-year-old female patient with significant history of polycystic liver disease who presented to the hospital with recurrent hematemesis and melena. She underwent esophagogastroduodenoscopy which showed multiple large esophageal varices requiring banding.

## 1. Introduction

Acute variceal bleeding is most commonly caused by portal hypertension secondary to liver cirrhosis. Polycystic liver disease is a rare etiology for portal hypertension and bleeding varices [1]. Portal hypertension is usually seen in advanced stages of polycystic liver disease. It can result from mechanical pressure of enlarging cysts on vascular structures such as hepatic veins, inferior vena cava, and portal vein or can be caused by secondary fibrosis in the setting of polycystic liver disease [1, 2]. We present a case of a 67 year old female patient with significant history of polycystic liver disease and had recurrent episodes of variceal bleeding requiring multiple banding.

## 2. Case Report

A 67-year-old Caucasian female patient presented to the hospital with one day history of hematemesis and melena. She had two episodes of bright red emesis and four episodes of dark colored stools the day she presented, followed by a brief syncopal episode lasting for 10-15 seconds. She also complained of epigastric pain. She was diagnosed with polycystic liver disease and portal hypertension one year ago when she presented similarly with hematemesis and melena. At that time, she had an esophagogastroduodenoscopy (EGD) which showed esophageal varices and underwent banding. She denied any history of alcohol use, NSAID ingestion, or peptic ulcer disease. She was gravida 3, para 3, and had tubal ligation following her last pregnancy. She denied use of oral contraceptive pills or hormonal replacement therapy in the past. Her family history was significant for cystic liver disease and chronic liver failure in mother and maternal aunt.

At the time of presentation, she was afebrile (temperature of 98.7°F), tachycardic (heart rate of 110 beats/min), tachypneic (respiratory rate of 18/min), and hypotensive (blood pressure of 97/60 mmHg). Cardiopulmonary examination was normal. Abdominal examination showed mildly distended abdomen with epigastric and right upper quadrant tenderness, and bowel sounds were normal. Extremities showed bilateral 1+ pitting edema. Initial laboratory work showed hemoglobin of 7.7 g/dl, hematocrit of 24%, white blood count of 10.8 cells/mm^3^, and platelet count of 162 cells/mm^3^. Metabolic panel showed nonanion gap metabolic acidosis with serum bicarbonate of 17 mEq/L, normal serum BUN, and creatinine of 23 mg/dl and 0.57 mg/dl, respectively. Liver panel showed total bilirubin of 2.2 mg/dl, direct bilirubin of 0.4 mg/dl, albumin of 2.5 g/dl, alkaline phosphatase of 51U/L, AST of 22 U/L, and ALT of 16 U/L. Gamma-glutamyltransferase was measured to be 27 U/L and prothrombin time (INR) was measured to be 1.46.

**Figure 1:**
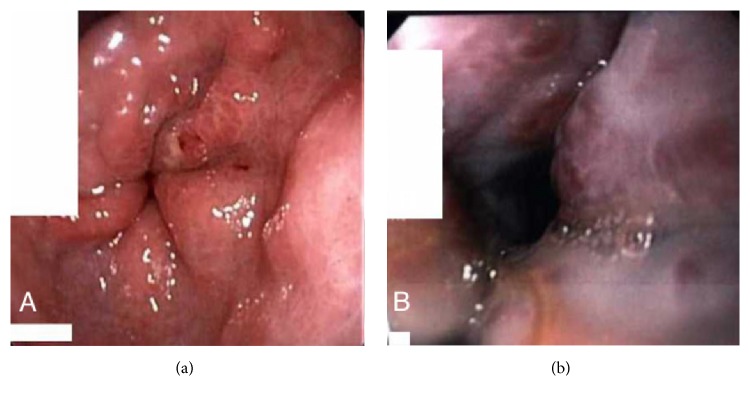
Endoscopy images showing large esophageal varices.

**Figure 2:**
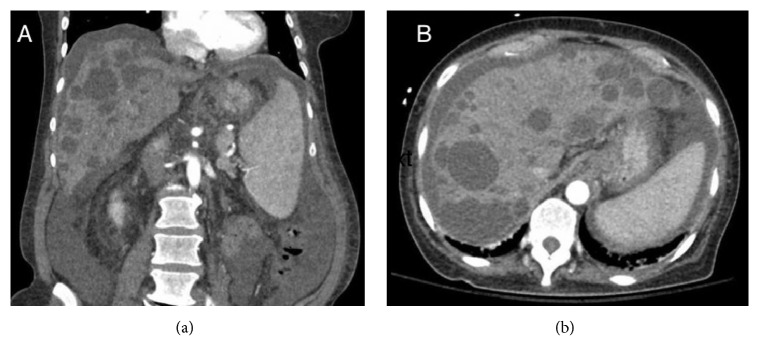
Computed tomography of abdomen and pelvis (coronal and axial scans) showing polycystic liver disease along with evidence of portal hypertension in form of splenomegaly and ascites.

**Figure 3:**
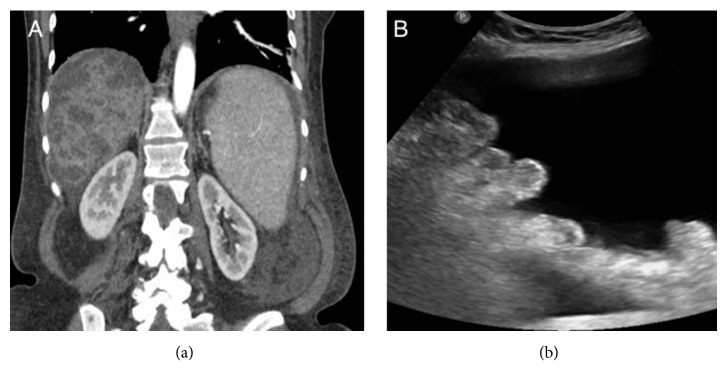
(a) Computed tomography of abdomen and pelvis (coronal view) showing normal architecture and enhancement of bilateral kidneys with no evidence of renal cysts. (b) Abdominal ultrasonographic image showed gross ascites.

She was aggressively resuscitated with fluids, pressors, and packed red blood cell (PRBC) transfusion. Octreotide and proton pump inhibitor infusions were started. Patient was electively intubated for hemodynamic instability and altered mental status. An EGD was done which showed four columns of large esophageal varices with red wale sign showing stigmata of high risk for bleeding and type 1 gastroesophageal varices (Figure 1). Four bands were successfully applied starting from cardia up to midesophagus. A computed tomography (CT) of abdomen showed multiple cysts in the liver with evidence of portal hypertension in form of splenomegaly, ascites, and esophageal varices (Figure 2). Liver surface was smooth and no thrombus was identified in hepatic veins and inferior vena cava. Bilateral kidneys were normal with no evidence of renal cysts (Figure 3(a)). Vital signs and hemoglobin were monitored closely. Patient was successfully extubated next day and pressors were weaned off. Patient did not have any further episode of hematemesis but continued to have melena. She slowly dropped her hemoglobin requiring transfusions with PRBC. An EGD was repeated which reveled 3 more columns of esophageal varices with intact bands from previous EGD. Four more bands were placed on remaining varices. Following repeat EGD patient's hemoglobin remained stable for rest of her hospital stay. During her hospital stay, she also underwent therapeutic paracentesis with removal of 330 ml of peritoneal fluid for worsening ascites (Figure 3(b)). Peritoneal fluid analysis showed serum ascites albumin gradient (SAAG) of more than 1.1 g/dl, normal white cell count, negative gram stain, and cultures. She was started on propranolol, furosemide, and spironolactone. She was discharged home with recommendations to follow up in outpatient clinic with a plan of repeat EGD in 3-4 weeks.

## 3. Discussion

Polycystic liver disease (PLD) is an inherited disorder characterized by development of multiple cysts in the liver. It can occur as an isolated disease or in combination with polycystic kidney disease. Most of the cases of isolated polycystic liver disease are inherited in autosomal dominant pattern and hence it is also referred to as autosomal dominant polycystic liver disease (ADPLD). Isolated PLD is a genetically heterogeneous disorder and in majority of cases (approximately 70-80%) no mutation is identified [2, 3]. In rest minority of the cases (approximately 20-30%) a mutation in either PRKCSH or SEC63 gene is identified [4, 5]. PRKCSH gene is located on short arm of chromosome 19 (19p13.2-13.1) and encodes for a protein called hepatocystin [4]. SEC63 gene located on long arm of chromosome 6 (6q21-q23) [5]. Both these genes encode for proteins which are located within endoplasmic reticulum and play important role in synthesis of glycoproteins. Isolated PLD is a rare with an estimated prevalence of 1/100000 to 1/1000000 [1]. However, this might be underestimation of the true prevalence, as most of the patients have asymptomatic disease and never get diagnosed. More commonly PLD occurs in combination with polycystic kidney disease which is inherited either as autosomal dominant (ADPKD) or as autosomal recessive (ARPKD). Both of them can have liver involvement in the form of formation of multiple cysts in liver.

Liver cysts arise due to malformation of the ductal plate during embryonic development of biliary tract in liver [3]. There is proliferation of biliary ductules which leads to formation of biliary microhamartomas, also called von Meyenburg complexes. These biliary microhamartomas can be subject to progressive dilatation, leading to formation of liver cysts. The extent of liver involvement in PLD varies from few cysts confined to one or more segments to massive cystic enlargement of liver diffusely involving all segments. The severity of disease is seen to be more in females especially with history of multiple pregnancies or prolonged exposure to exogenous estrogen. Females tend to present at early age, have more widespread disease, and are more likely to progress to liver failure [2].

Majority of patients with isolated PLD (more than 80%) are asymptomatic with detection of liver cysts an incidental finding [3]. Minority of the patients develop symptoms which are typically secondary to enlarging size and mass effect of liver cysts. These include abdominal fullness, abdominal discomfort, back pain, anorexia, early satiety, malnutrition, gastroesophageal reflux, and dyspnea. Sometimes, it may present with acute abdominal pain which is secondary to cyst rupture, hemorrhage, or infection. Very rarely, patients of PLD can present with signs and symptoms of portal hypertension as seen in our patient. Portal hypertension is usually seen in advanced stages of PLD and can be caused by a number of processes in polycystic liver disease [1]. Firstly, portal hypertension can result from mechanical pressure from enlarging cysts on hepatic veins or inferior vena cava leading to obstruction in hepatic vein outflow. In some cases of polycystic liver disease, hepatic vein outflow obstruction may occur secondary to hepatic vein thrombosis (Budd-Chiari syndrome) [1, 6]. Secondly, enlarging hepatic cysts can compress on portal vein inflow leading to portal hypertension. Lastly, in advanced stages of disease there may be development of liver fibrosis which might lead to portal hypertension [1]. Portal hypertension leads to development of esophageal varices, splenomegaly, and ascites. Jaundice is an uncommon finding in polycystic liver disease and seen only in advanced stages. Liver functions are usually preserved in majority of patients of PLD and progression to end stage liver disease is rare. The most common laboratory abnormality seen in polycystic liver disease is mild elevation of gamma-glutamyltransferase and alkaline phosphatase [2].

Diagnosis of PLD is established radiologically by imaging studies including ultrasonography, CT, and magnetic resonance imaging. Treatment strategies for PLD include conservative management, invasive approach, and medical therapy [7]. Long-term outcome in majority of patients of PLD is good with normal quality of life. These patients if asymptomatic will require no treatment. In those who are symptomatic or have advance disease, various invasive procedures can be used for management of PLD and include aspiration sclerotherapy, laparoscopic fenestration, surgical deroofing, and segmental hepatic resection [7]. Liver transplantation is considered in patients who suffer tremendous morbidity and medical complications from severe PLD and are unsuitable for segmental hepatic resection or surgical interventions. Although no medical therapy is presently approved for treatment of PLD, recent studies with somatostatin analogues and mTOR inhibitors have been encouraging. When compared to placebo both octreotide and lanreotide (somatostatin analogues) have shown significant reduction in total liver volume in patients with PLD [8, 9]. Similarly, use of sirolimus and everolimus (mTOR inhibitors) has shown significant reduction in polycystic liver volumes suggesting a potential role in the treatment of PLD [10]. However further studies are needed to determine their long-term efficacy and tolerability in PLD. As discussed earlier, development of portal hypertension in PLD may be multifactorial and can be of significant concern in advanced disease. Portal hypertension and its complications such as acute variceal bleeding and recurrent ascites can be difficult to treat in patients of PLD. Somatostatin analogues can potentially serve as useful medical treatment in this regard due to their dual effect on decreasing the portal venous pressure and arresting the variceal bleeding as well reducing the cyst size and total liver volume. Currently, patients of PLD with recurrent complications of portal hypertension are treated with surgical portosystemic shunt creation and/or liver transplantation.

Polycystic liver disease has been considered a contraindication for transjugular intrahepatic portosystemic shunt (TIPPS) creation. Although, the rationale behind this is poorly documented, it is believed that presence of multiple cysts in liver leads to hepatic architectural distortion causing technical difficulties in creation of TIPPS. However, the absolute contraindication of TIPPS in polycystic liver disease has been challenged recently in a number of case reports [11–14]. These case reports have documented successful creation of TIPPS in polycystic liver disease patients warranting reconsideration of this contraindication.

## 4. Conclusion

Isolated polycystic liver disease is a rare disease and portal hypertension presenting as acute variceal bleeding is an uncommon manifestation of it.
